# Elevated body mass index is associated with delayed protective airway mucosal immune responses in mild SARS-CoV-2 infection

**DOI:** 10.1016/j.ebiom.2026.106215

**Published:** 2026-03-27

**Authors:** Joe Fenn, Rhia Kundu, Janakan Sam Narean, Emily Conibear, Aleksandra Koycheva, Seran Hakki, Nieves Derqui, Sean Nevin, Mica Tolosa-Wright, Jakob Jonnerby, Kieran Madon, Samuel Baldwin, Timesh D. Pillay, Robert Varro, Constanta Luca, Graham Taylor, Jake Dunning, John S. Tregoning, Ajit Lalvani, INSTINCT Study Investigators, Lulu Wang, Anjna Badhan, Eleanor Parker, Carolina Rosadas, Lucy G. Mosscrop, Patricia Watber, Jie Zhou, Jack L. Barnett, Hamish Houston, Anika Singanayagam, Paul S. Freemont, Neil M. Ferguson, Maria Zambon, Wendy S. Barclay, Richard Tedder, Myra McClure, Jessica Cutajar, Valerie Quinn, Sarah Hammett, Eimèar McDermott, Kristel Timcang, Jada Samuel, Samuel Bremang, Samuel Evetts, Megan Davies, Chitra Tejpal, Anjeli V. Ketkar, Giulia Miserocchi, Harriet Catchpole, Simon Dustan, Isaac J. Day Weber, Federica Marchesin, Alexandra Kondratiuk

**Affiliations:** a NIHR Health Protection Research Unit in Respiratory Infections, Imperial College London, London, UK; b National Heart and Lung Institute, Imperial College London, London, UK; c MRC Centre for Global Infectious Disease Analysis, School of Public Health, Imperial College London, UK; d Department of Infectious Disease, Imperial College London, London; e Pandemic Sciences Institute, University of Oxford, Oxford, UK

**Keywords:** COVID-19, Mucosal immunity, Cytokines, Immunoglobulin A, Body mass index

## Abstract

**Background:**

SARS-CoV-2 viral load in the upper respiratory tract (URT) typically peaks and declines within days of infection, even in individuals without prior infection or vaccination. Although this implicates the URT innate immune response in effectively restricting viral replication, the nature of the protective responses and how they are affected by demographic factors is poorly defined.

**Methods:**

We recruited 54 seronegative household contacts of recently diagnosed COVID-19 cases and prospectively collected URT samples during and after exposure. Among the 39 individuals who became infected, we quantified airway mucosal cytokine and chemokine responses and virus-specific nasal IgA using Meso Scale Discovery assays, and assessed associations with demographic factors, viral load, and symptoms.

**Findings:**

Participants with higher BMI had higher URT viral loads and more marked symptoms. This was significantly associated with delayed induction of protective inflammatory mediators in the airway mucosa but not in blood. Induction of virus-specific nasal IgA at 1-week post-infection also correlated with lower viral load.

**Interpretation:**

Elevated BMI retards initial airway mucosal innate immune responses to infection, which may partially explain the pronounced adverse impact of higher BMI on clinical and virological outcomes in COVID-19.

**Funding:**

This work is supported by the NIHR Health Protection Research Unit in Respiratory Infections, Imperial College London in partnership with the UK Health Security Agency (Grant number: NIHR200927; AL) and the 10.13039/501100000265Medical Research Council (Grant number: MR/X004058/1). Infrastructure support for this research was provided by the 10.13039/501100013342NIHR Imperial Biomedical Research Centre (BRC).

**Research in context**

**Evidence before this study**

A search of PubMed for manuscripts published in English between 2019 and August 2025 using the term (“SARS-CoV-2” OR “COVID-19”) AND (“Mucosal”) AND (“Cytokine” OR “Chemokine”) AND (“Human” OR “cohort”) returned 83 results. Evidence in these manuscripts shows that upper respiratory tract (URT) immune responses play a pivotal role in controlling respiratory viral replication. Human studies and experimental challenge models indicate that early induction of type I and III interferons in the URT is associated with milder disease, whereas delayed interferon activity alongside prolonged elevation of pro-inflammatory cytokines such as IL-6, CXCL10, and CXCL8 in both the URT and peripherally correlates with worse outcomes. Demographic factors including older age, male sex, and obesity are well established risk factors for severe COVID-19, yet how these influence early mucosal antiviral responses in the URT remains poorly defined.

**Added value of this study**

High Body Mass Index (BMI) is an established risk factor for more severe COVID-19 outcomes, but the mechanisms are poorly understood. By recruiting unvaccinated household contacts of recent COVID-19 cases we were able to characterise early post-exposure airway mucosal immune responses in the upper respiratory tract (URT) in the absence of preexisting adaptive immunity. In newly infected contacts, early airway mucosal chemokine and cytokine concentrations were associated with lower viral loads. However, individuals with higher BMI exhibited a striking delay in induction of these protective nasal responses despite normal onset of systemic responses. Nasal IgA production also correlated with lower viral load and was associated with nasal cytokine and chemokine responses.

**Implications of all the available evidence**

Airway mucosal cytokine and chemokine responses have a protective role in SARS-CoV-2 infection, and their temporal regulation is a key factor in their relationship with clinical and virological outcomes of infection as well as nasal antibody induction. High BMI, which is a well-established risk factor for more severe COVID-19, may affect clinical outcome by retarding protective early mucosal immune responses, highlighting the potential of therapies that enhance URT innate responses in at-risk individuals. The fact that even modestly elevated BMI, below the threshold for obesity, was associated with markedly slower induction of mucosal innate immune responses suggests that this effect has broad public health implications at the population level.

## Introduction

Demographic associations with SARS-CoV-2 infection outcome are well established. Elevated Body Mass Index (BMI) and advanced age correlate strongly with poorer clinical outcomes of infection.^1^^,^^2^ Even modestly elevated BMI can influence COVID-19 outcome, with each additional BMI unit above 23 kg/m^2^ having been shown to increase risk of hospital admission by ∼5% and each additional unit above 28 increasing risk of death by 4%.^2^ In addition to association with COVID-19 outcomes, age and BMI have been shown to affect the magnitude of both steady state and respiratory pathogen-induced immune responses.^3^^,^^4^

It has been hypothesised that the strong association between high BMI and poor clinical outcomes to COVID-19 is caused, in part, by immunomodulatory effects of being overweight.^5^ Indeed, cytokines associated with a chronic inflammatory state, including TNF-α and IL-6, are constitutively elevated in those with a high BMI and dysregulation of the same proteins during SARS-CoV-2 infection is a predictor of poor COVID-19 clinical outcome.^6^^,^^7^ The relationship between airway mucosal cytokine production and clinical and virological outcomes of COVID-19 is less well studied. The dynamics of airway mucosal immune factors have been well characterised in controlled human infection model (CHIM) studies^8^ but these have been limited to healthy, fit volunteers without elevated BMI. Nasal anti-SARS-CoV-2 antibodies are also candidate mediators of improved control of SARS-CoV-2 viral load; IgA is the main contributor to SARS-CoV-2 neutralisation in the airway mucosa.^9^ In this study, we therefore investigated relationships between BMI and early upper respiratory tract (URT) cytokine, chemokine and IgA responses and assessed the impact of these on clinical and virological outcomes of mild COVID-19.

To identify key host factors mediating optimal viral control, it is necessary to investigate immune responses at the site of initial exposure, the URT mucosa, during the very first days of infection. We collected nasal lining fluid (NLF) and other biological samples from household contacts of COVID-19 cases within 3 days of well-defined SARS-CoV-2 exposure and longitudinally thereafter. We characterised cytokines and nasal IgA in the URT throughout the course of infection and investigated correlates of improved viral control. In parallel we investigated the impact of BMI, on both virological outcomes and early immune responses. To exclude the confounding effects of pre-existing adaptive immunity all contacts included in this study were unvaccinated and had no prior SARS-CoV-2 infections. Understanding how increasing BMI affects these early immune responses could provide insight into the mechanisms through which it increases disease severity as the suppression of viral replication in the URT within 2–3 days of infection that occurs in most immunologically naïve individuals is likely mediated by early innate mucosal responses at the site of infection.^10^

## Methods

### INSTINCT study design and recruitment

INSTINCT (Integrated Network for Surveillance, Trials and Investigations into COVID-19 Transmission) was a prospective longitudinal study of COVID-19 cases and their household contacts that recruited a total of 383 participants from 139 households between May 2020 and March 2021. Briefly, SARS-CoV-2 positive index cases were identified through Public Health England (PHE) (now UK Health Security Agency (UKHSA)) and NHS National Test and Trace (NTAT) programme in the United Kingdom. Index cases that were referred to the study team within 5 days of symptom onset were enrolled as well as all their household contacts that consented to study enrolment. Research nurses visited study participants on the day of enrolment (day 0) and days 7, 14, and 28 post enrolment. During study visits, blood, nasal lining fluid and nose and throat swabs were collected. An additional nose and throat swab was self-collected by participants on day 4, as previously described (REC Reference: 20/NW/0231 IRAS: 282820).^11^ Participants gave written informed consent and were free to leave the study at any time.

### Sample schedule and processing

Participants provided nose and throat swabs at the baseline and study days 4, 7, 14 and 28. RT-PCR targeting SARS-CoV-2 E gene was used to quantify SARS-CoV-2 viral load as previously described.^12^ Blood and nasal lining fluid (NLF) were collected at baseline, day 7 and day 28 visits. Double Antigen Binding Assay (DABA) was used to quantify blood SARS-CoV-2 anti-S RBD antibody as previously described.^13^^,^^14^ Results of this assay, in combination with PCR, were used to assign participant infection status. Research nurses visited participant's homes and collected venous blood samples and NLF. Serum was isolated by centrifugation of whole blood collected in red-top tubes after clot formation and was frozen at −80 °C until processing. To collect NLF, nurses placed a synthetic absorptive matrix (SAM) strip (Hunt Developments UK Ltd; Cat No. FX·i) in the participant's nostril and gently pinched the nostril closed onto the strip for 1 min. The strip was transported on ice and frozen at −80 °C until processing at BSL3. Elution was performed as previously described and samples were inactivated with 1% Triton X-100 (Sigma; Cat No. X100) prior to downstream analysis.^15^

### Symptom diary and scoring

Participants completed a daily symptom questionnaire for 28 days post-enrolment assessing the presence or absence of 20 symptoms. Questionnaires were used to derive scores for symptom burden, upper respiratory tract-associated symptoms and systemic symptoms (Table S1).

### Cohort description

Of 383 INSTINCT participants 245 were contacts. 205 of these had a PCR or serologically confirmed COVID-19 index case. We excluded 44 contacts that were SARS-CoV-2 seropositive at enrolment and 38 from whom serial PCR data was not available. 28 contacts were excluded from further analysis due to a lack of available serial serum, or NLF samples. Therefore, samples from a total of 95 individuals collected between November 2020 and April 2021 were included in this study. Contacts were distributed between 60 individual households (1 Household with 5, 9 households with 3, 12 households with 2 and 38 households with 1 contact).

The day on which infected contacts first became PCR-positive varied between individuals. To adjust for the effect of this on downstream analyses, samples were categorised based on the day on which the first PCR-positive swab was collected. As such, ‘study days’ were converted to ‘infection timepoints’. FP corresponds to the timepoint at which an individual first becomes PCR-positive, FP + 4 denotes 4 days after FP and FP + 7 denotes 7 days after FP. Note that where donors first became PCR-positive on study d4, study day 7 was considered FP + 4 as opposed to FP + 3—this was a pragmatic choice to enable samples to be aligned for downstream analyses. Demographic characteristics including biological sex were self-reported by participants and were included as descriptive variables in the study dataset.

### Meso scale discovery cytokine profiling

CCL2, CCL3, CCL4, CCL8, CXCL10, G-CSF, GM-CSF, IFNα2, IFNβ, IFNγ, IFNλ, IL-10, IL-18, IL-1RA, IL-1β, IL-27, IL-4, IL-6, IL-8 and TNFα were quantified using a custom U-plex panel and MSD U-Plex plates (Mesoscale Diagnostics (MSD), Rockville, Maryland, USA) as per manufacturer's instructions. Assay diluent blanks were included to estimate and subtract background signal, full standard curves with QC controls were run on each plate, and all samples were assayed in technical duplicates. Concentrations were calculated from background-subtracted electrochemiluminescence signals using the standard curve, and values falling below the lower limit of detection were assigned a value of zero. Plates were read using a SQ120 Quickplex instrument (MSD; Cat No: AI1AA).

### Meso scale discovery IgA profiling

HCoV-229E Spike, HCoV-NL63 Spike, HCoV-OC43 Spike, HCoV1-HKU1 Spike, SARS-CoV-1 Spike, SARS-CoV-2 Nucleocapsid, SARS-CoV-2 Spike, SARS-CoV-2 S1 NTD, and SARS-CoV-2 S1 RBD were quantified using MSD V-PLEX COVID-19 Coronavirus Panel 2 (IgA) kit (MSD; Cat No: K15371U) as per manufacturer's instructions. Plates were read using a SQ120 Quickplex instrument (MSD; Cat No: AI1AA).

### Statistical analyses

All analyses were conducted in GraphPad Prism v10. Consistent with our pre-specified analysis plan and policy for outliers, all data points were retained unless there was clear evidence of measurement error. Viral load was analysed on the log 10 scale (E-gene copies per ml), and sampling was indexed to infection timepoints (for example, FP, FP + 7, FP + 14, convalescence). Mann–Whitney U tests were used for comparisons between two groups (for example, PCR-positive vs PCR-negative; BMI < 25 vs BMI ≥ 25 at matched timepoints), Where multiple-Mann-Whitney U-tests were used to assess differences in multiple variables between groups (for example, a panel of cytokines in PCR-positive compared to PCR-negative contacts) with false-discovery rate correction using the two-stage Benjamini–Krieger–Yekutieli procedure at q = 0.05, and volcano plots display mean rank differences as the effect metric. Correlations between cytokines/chemokines, nasal IgA, viral load, symptom burden, and demographics (age, BMI) were assessed using Spearman's rank correlation at specified timepoints without corrections for multiple comparisons unless otherwise stated.

Mixed-effects analysis with Geisser-Greenhouse correction was used with Tukey's post-hoc correction for multiple comparisons of repeated-measures data with missing data, for example analysis of cytokine concentrations over time. The model uses the restricted maximum likelihood (REML) method and defines initial values based on the general linear model (GLM) and makes no assumptions of sphericity. QQ (Quantile–Quantile) residuals plots were visually inspected to confirm that residuals were approximately normally distributed. Missing values were not imputed; analyses used all available observations at each timepoint, with the mixed-effects framework accommodating unbalanced repeated measures. Kruskal–Wallis tests with Dunn's multiple comparisons were used for multi-timepoint IgA data as this was assumed to be non-normally distributed. Proportions were compared using Fisher's exact test with reporting of relative risk and exact 95% confidence intervals.

### Ethics

Ethical approval for this study was obtained from the Northwest–Greater Manchester East Research Ethics Committee in accordance with Health Research Authority regulations (REC Reference: 20/NW/0231 IRAS: 282820). All participants were fully informed about the study's objectives, procedures, potential risks, and benefits and provided written informed consent prior to their inclusion in the study.

### Role of funders

Study funders had no role in study design, data collection, data analyses, interpretation, or writing of this report.

## Results

### Elevated BMI is associated with a protracted viral load trajectory and higher symptom burden in mild COVID-19 cases and higher likelihood of infection in exposed contacts

**Fig. 1:**
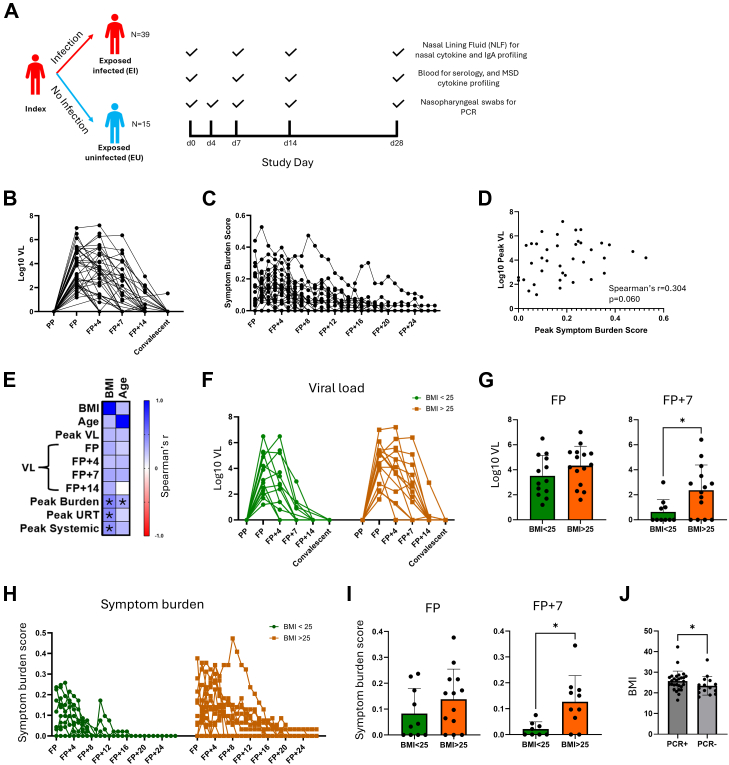
Elevated BMI associated with delayed viral clearance and higher symptom burden. A. Graphical representation of study sampling schedule. Household contacts of PCR-confirmed COVID-19 index cases were recruited and the indicated biological specimens were collected longitudinally at study days 0, 4, 7, 14 and 28. n values represent number of participants from whom nasal cytokine concentrations were measured. **B.** Dynamics of viral loads (VL) in PCR-positive contacts (n = 39). VL is presented as log 10 E gene copy numbers. Sampling timepoints are adjusted to ‘Infection timepoints’ by adjusting to day of collection of first PCR-positive sample. PP = Pre-PCR positive time point, FP = first PCR-positive time point; FP + 7 = 7 days post-FP; FP + 14 = 14 days post-FP; convalescent = 21 or 28 days post-FP. Note, d0 PCR data for one individual is absent due to human error in sample handing. Assignment of d0 samples from this individual to the FP time point was based on symptom data. **C.** Dynamics of symptom burden score in PCR-positive contacts. **D.** Correlation between peak VL and peak symptom burden score (n = 39, Spearman's r = 0.304 p = 0.060). **E.** Spearman's correlation matrices of associations between BMI, age and the indicated viral load and symptom burden outcomes of infection. Colours represent Spearman's r values. Positive correlations are denoted by blue, negative correlations are denoted by red. Correlations with unadjusted p < 0.05 are indicated with an asterisk. **F.** Viral load (Log 10 E gene copy number/ml) trajectories over time in contacts with BMI < 25 (green) and contacts with BMI > 25 (orange) **G.** Viral loads compared between contacts with BMI < 25 (green) and contacts with BMI > 25 (orange) at FP and FP + 7 using Mann Whitney U test. ∗p < 0.05. **H.** Symptom burden score trajectories over time in contacts with BMI < 25 (green) and contacts with BMI > 25 (orange). **I.** Symptom burden scores compared between contacts with BMI < 25 (green) and contacts with BMI > 25 (orange) at FP and FP + 7 using Mann Whitney U test. ∗p < 0.05. **J.** BMI of PCR-positive contacts compared to PCR-negative contacts using Mann Whitney U test (p = 0.0481).

Nasal lining fluid (NLF), nose and throat upper respiratory tract (URT) swabs, and blood samples were collected from 54 household contacts of recently infected community COVID-19 cases during exposure and longitudinally thereafter. PCR was performed on URT swabs and serology was performed on blood to determine infection status. 39 of 54 contacts became PCR-positive, while 15 remained serially PCR-negative and seronegative (demographic data available in Table S2). The 39 PCR-positive contacts were distributed between 26 households (3 households with 3 contacts, 7 households with 2 contacts and 16 households with 1 contact). Airway mucosal and systemic cytokine and antibody profiling was performed as indicated (Fig. 1A).

The day on which infected contacts first become PCR-positive varied between individuals. To adjust for the effect of this on downstream analyses, samples were categorised based on the day on which the first PCR-positive swab was collected. As such, ‘study days’ were converted to ‘infection timepoints’. For example, if an individual was PCR-positive at enrolment, i.e. study day 0, samples collected at this time were assigned to the ‘first positive’ (FP) infection timepoint. If an individual first became PCR-positive on study day 7, the study day 0 samples collected prior to acquisition of PCR-detectable infection were assigned to the ‘pre-positive’ (PP) infection timepoint and the corresponding study day 7 samples assigned to the FP infection timepoint. The first positive PCR result (FP) occurred at d0 in 32 participants, d4 in 6 participants and d7 in 1 participant. (d0 PCR data was missing for one individual. In this case FP was assigned to d0 based on symptom data).

Peak viral load (pVL) varied between individuals (Mean = 4.08, SD = 1.59 (log 10 E gene copies/ml)) as did viral load trajectory (Fig. 1B). Clinical parameters were recorded daily by participants and quantified as a daily Symptom Burden Score (SBS), a composite score of presence and severity of 20 symptoms (Table S1). Like pVL, peak SBS (pSBS) varied considerably between individuals (Mean = 0.188, SD = 0.128) (Fig. 1C). pVL did not correlate significantly with pSBS (p = 0.060) (Fig. 1D).

**Figure S1:**
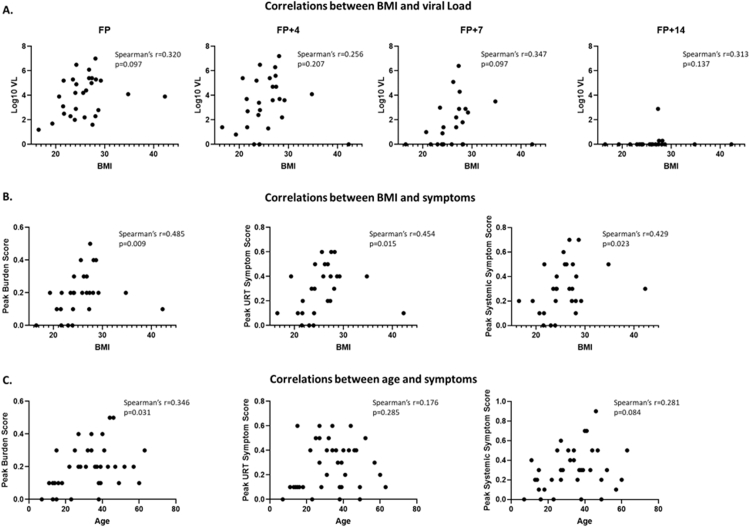
**Correlations between BMI, age and virological and clinical outcomes**. Individual X Y plots for correlations displayed in the heatmap shown in Fig. 1E with Spearman’s r and associated p values displayed. A. Correlations between BMI and virological outcomes. B. Corelations between BMI and clinical outcomes. C. Correlations between age and clinical outcomes.

Both BMI and age are well established demographic correlates of COVID-19 outcomes, with high BMI and advanced age strongly associating with more severe clinical outcomes of infection. In our cohort of mild COVID-19 cases, both age and BMI positively correlated with symptom burden score (Fig. 1E). BMI did not correlate significantly with viral load at any timepoint, though correlations approached statistical significance at FP and FP + 7 (p = 0.0969 and p = 0.0967 respectively, Figure S1). To further investigate associations between BMI and virological outcomes, infected contacts were stratified by BMI using a threshold of 25 based on UK Health Security Agency (UKHSA) and US Centre for Disease Control (CDC) classification of BMI > 25 as overweight and viral load trajectories plotted (Fig. 1F). VL was not significantly different between BMI groups at FP but was significantly higher in the BMI > 25 group at FP + 7 suggesting a longer duration of infection in those with elevated BMI (Fig. 1G). A similar pattern was observed in symptom burden trajectories (Fig. 1H). Whilst symptom score was similar between BMI groups at FP, there was a significantly higher symptom burden score in the BMI > 25 group at FP + 7 (Fig. 1I). Together, our data support an association between elevated BMI and impaired control of viral replication paired with greater burden of disease.

### Elevated BMI is associated with increased susceptibility to infection in exposed contacts

**Figure S2:**
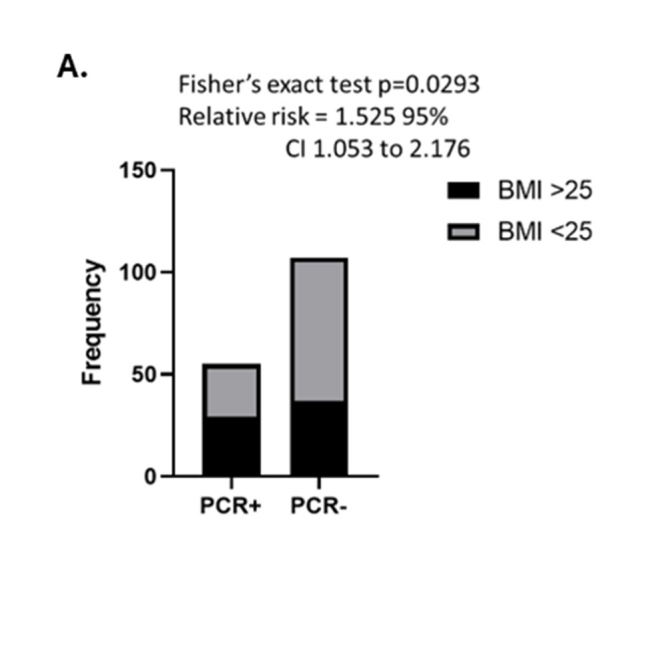
**Relationship between BMI and risk of infection across the entire INSTINCT cohort**. **A.** Frequency of PCR-positive and PCR-negative contacts with BMI values above and below a threshold of 25. Fisher’s exact test was performed to determine differences in BMI group distributions between infected and uninfected contacts. Fisher’s exact test Relative Risk 1.525, 95% CI 1.053–2.176, p = 0.0293.

BMI was also significantly higher in infected contacts compared to uninfected contacts (Fig. 1J). The association between high BMI and risk of infection was investigated in the wider INSTINCT cohort including individuals in whom nasal cytokine analysis could not be performed. Of 162 recently exposed COVID-19 contacts with available PCR and serology data, those with a BMI > 25 were at significantly higher risk of being PCR-positive (Figure S2).

### Nasal cytokine responses correlate with COVID-19 outcome and are retarded in those with elevated BMI

**Fig. 2:**
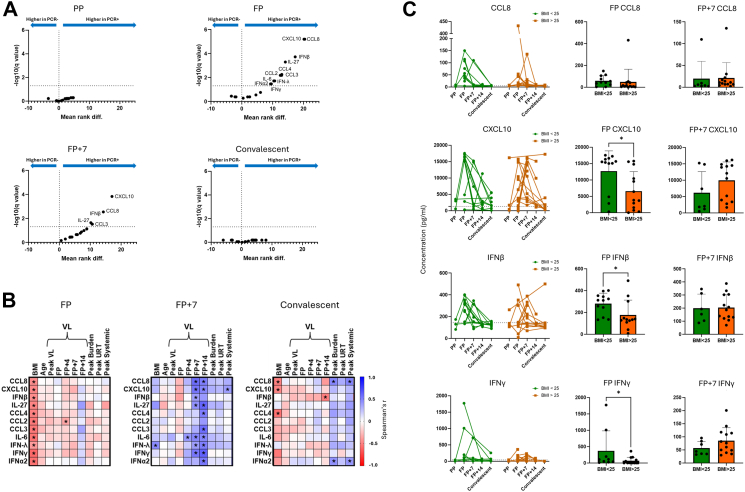
BMI negatively correlates with early nasal cytokine response and influences SARS-CoV-2 viral control. **A.** Volcano plots depicting mean ranked difference outputs of multiple Mann Whitney U tests with two-stage step-up Benjamini, Krieger and Yekutieli FDR cut-off of 5% comparing concentrations of 20 cytokines in nasal lining fluid (NLF) between PCR-positive and PCR-negative groups. Both PP and FP infection timepoint samples from PCR-positive samples were compared to d0 samples from PCR-negative contacts because the majority of FP samples from PCR-positive cases were collected at d0. Accordingly, FP + 7 samples from PCR-positive contacts were compared to d7 samples from PCR-negative contacts and convalescent samples from PCR-positive contacts were compared to d28 samples from PCR-negative contacts. (n = 15 PCR-negative, 39 PCR-positive). Analytes that were significantly different between groups (q < 0.05) are annotated. **B.** Spearman's correlation matrices of correlations between nasal lining fluid concentrations of infection-associated cytokines (defined in Fig. 2A) and the indicated demographic factors and outcome measures in PCR-positive contacts at the indicated time points. Colours represent unadjusted Spearman's r values. Positive correlations are denoted by blue, negative correlations are denoted by red. ∗Unadjusted p < 0.05. Sample availability at each timepoint is displayed in Figure S5. **C.** PCR-positive contacts were categorised into two BMI groups (BMI < 25 and BMI > 25). Nasal cytokine trajectories were plotted over time, with green lines representing contacts with BMI < 25 and orange lines representing those with BMI > 25. Depicted are analyses of all cytokines that were significantly elevated at FP or FP + 7 relative to other timepoints and most highly upregulated in PCR-positive contacts (Figure S2 + Fig. 3A). Differences in cytokine expression between BMI groups at FP and FP + 7 were assessed using the Mann–Whitney U test. ∗p < 0.05.

**Figure S3:**
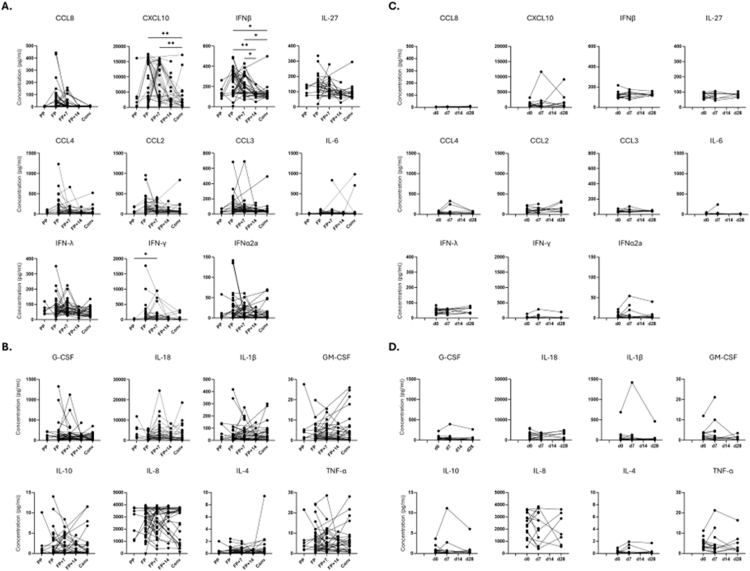
**Nasal cytokine dynamics in PCR-positive and PCR-negative contacts**. Change in nasal cytokine concentration over time in PCR-positive and PCR-negative contacts. Asterisks denote significant differences between time points according to Mixed effects analysis with Tukey’s test for multiple comparisons. ∗p < 0.05, ∗∗ p < 0.01, ∗∗∗p < 0.001, ∗∗∗∗p < 0.0001. **A.** Expression in PCR-positive contacts of cytokines that were differentially expressed between PCR-positive and PCR-negative contacts. **B.** Expression in PCR-positive contacts of cytokines that were not significantly differentially expressed between PCR-positive and PCR-negative contacts. **C.** Expression in PCR-negative contacts of cytokines that were differentially expressed between PCR-positive and PCR-negative contacts. **D.** Expression in PCR-negative contacts of cytokines that were not significantly differentially expressed between PCR-positive and PCR-negative contacts.

Our household contact study design enables investigation of relationships between very early immune responses and infection outcomes. Initial samples were generally collected from contacts at around symptom onset (Mean 0.18, days from first reported symptom to enrolment, SD 2.70). 11 of the 20 measured cytokines and chemokines were significantly upregulated in PCR-positive contacts at FP compared to PCR-negative contacts at d0. (IFNα2, IFNβ, IFNγ, IFNλ, IL-6, IL-27, CCL2, CCL3, CCL4, CCL8, and CXCL10) (Fig. 2A). Of these, IFNβ, IL-27, CCL3, CCL8, and CXCL10 remained significantly elevated at FP + 7 in PCR-positive contacts relative to d7 samples from PCR-negative contacts. No nasal cytokines were differentially expressed between infected and uninfected contacts at convalescence. The dynamics of nasal cytokine production varied between individuals. CXCL10 was significantly upregulated at both FP and FP + 7 compared to convalescence, as was IFNβ (adjusted p = 0.0041, p = 0.0048, p = 0.0103, p = 0.0277 respectively) (Figure S3). IFNγ was significantly upregulated only at FP + 7 (adjusted p = 0.0234) (Figure S3). There were no significant differences in nasal cytokine concentration over time in PCR-negative contacts (Figure S3C and D).

**Figure S4:**
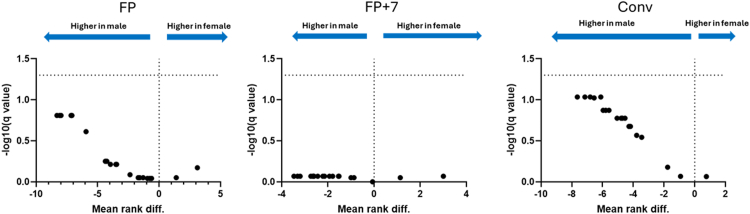
**Association of biological sex and nasal cytokine response to SARS-CoV-2 infection**. Concentrations of infection-associated cytokines were compared between biological males and females using Mann Whitney U tests with two-stage step-up Benjamini, Krieger and Yekutieli FDR cut-off of 5%. Mean ranked differences between groups are displayed in volcano plots.

Having identified 11 cytokines that were significantly higher in the airway mucosa during SARS-CoV-2 infection, we next examined how their concentrations related to clinical and virological outcomes. Given our observed associations between both age and BMI these demographic factors and infection outcomes, we also investigated the impact of these demographic factors on mucosal immune responses. Nasal cytokine concentrations at FP in PCR-positive contacts generally correlated negatively with viral load and symptom burden score though most associations were non-significant A significant negative correlation was, however, observed between CCL2 and subsequent viral load (FP + 4) (Fig. 2B). At the FP + 7 timepoint higher nasal cytokine concentrations associated with worse virological and clinical outcomes. A significant positive correlation between multiple nasal cytokines and both FP + 7 and FP + 14 viral load was observed, likely reflecting ongoing viral infection driving continued production of nasal cytokines. CXCL10 also correlated positively with peak systemic symptom score at FP + 7 (Fig. 2B). Strikingly, concentrations of all 11 infection-associated nasal cytokines correlated negatively with BMI at the earliest stage of infection (FP). This association was not observed 7 days later (FP + 7) (Fig. 2B). Unlike BMI, age did not significantly correlate with concentration of any infection-associated nasal cytokine at any time point (Fig. 2B). There were no significant differences in nasal cytokine production between biological sexes at any timepoint (Figure S4). Biological sex was self-reported by participants. These observations were not explainable by BMI-associated comorbidities or intake of immunomodulatory drugs. One individual in each of the BMI < 25 and BMI > 25 groups reported a comorbidity: in both cases asthma. No participants reported use of systemic corticosteroids or biologic immunomodulators.

To further investigate the relationship between BMI and early nasal cytokine responses, participants were stratified into two BMI groups using the threshold of 25 and cytokine dynamics were compared between these groups. We focused on mediators that were both significantly upregulated in PCR-positive contacts and showed significant changes over time. This approach was intended to maximise our ability to detect BMI-related differences, by focussing on cytokines that were commonly induced across participants and exhibited the greatest dynamic range in expression across time points (Figure S1). At FP, CXCL10, IFNβ and IFNγ were all significantly lower in the BMI > 25 group but cytokine production was not significantly different between groups at FP + 7 (Fig. 2C).

**Fig. 3:**
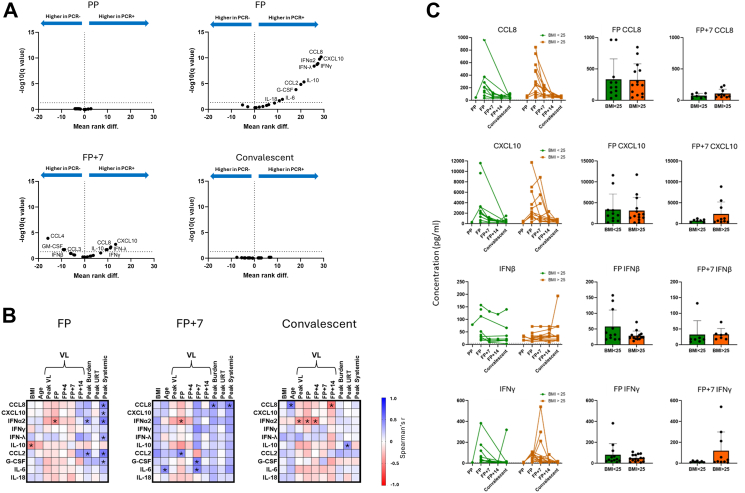
Serum cytokine response does not generally correlate with BMI but positively correlates with symptom burden. **A.** Volcano plots depicting mean ranked difference outputs of multiple Mann Whitney U tests with two-stage step-up Benjamini, Krieger and Yekutieli FDR cut-off of 5% comparing concentrations of 20 cytokines in serum between PCR-positive (n = 36) and PCR-negative (n = 32) groups. Both PP and FP infection timepoint samples from PCR-positive samples were compared to d0 samples from PCR-negative contacts because the majority of FP samples from PCR-positive cases were collected at d0. Accordingly, FP + 7 samples from PCR-positive contacts were compared to d7 samples from PCR-negative contacts and convalescent samples from PCR-positive contacts were compared to d28 samples from PCR-negative contacts. Analytes that were significantly different between groups (q < 0.05) are annotated. Figure S5 displays overlap in participants with available serum and nasal cytokine data). **B.** Spearman's correlation matrices of correlations between serum concentrations of infection-associated cytokines (defined in Fig. 4A) and the indicated demographic factors and outcome measures in PCR-positive contacts at the indicated time points. Colours represent Spearman's r values. Positive correlations are denoted by blue, negative correlations are denoted by red. ∗Unadjusted p < 0.05. Sample availability at each timepoint is displayed in Figure S5. **C.** PCR-positive contacts were categorised into two BMI groups (BMI < 25 and BMI > 25). Serum cytokine trajectories were plotted over time, with green lines representing contacts with BMI < 25 and orange lines representing those with BMI > 25. Depicted are analyses of the same cytokines selected for analysis in NLF in Fig. 3C. Differences in cytokine expression between BMI groups at FP and FP + 7 were assessed using the Mann–Whitney U test. ∗p < 0.05.

**Figure S5:**
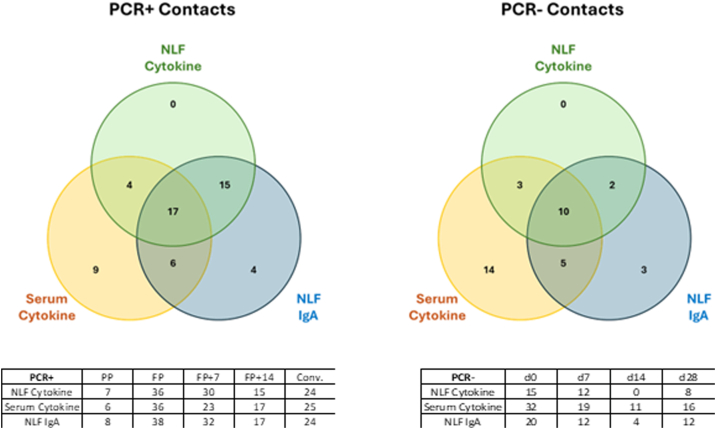
**Data availability across sample types and time points**. Venn diagram displaying the number of INSTINCT contacts with NLF cytokine, Serum cytokine and NLF IgA data available. Intersections between the circles represents frequency of contacts that are common to multiple data sets. Tables represent availability of data for each assay at individual timepoints.

**Figure S6:**
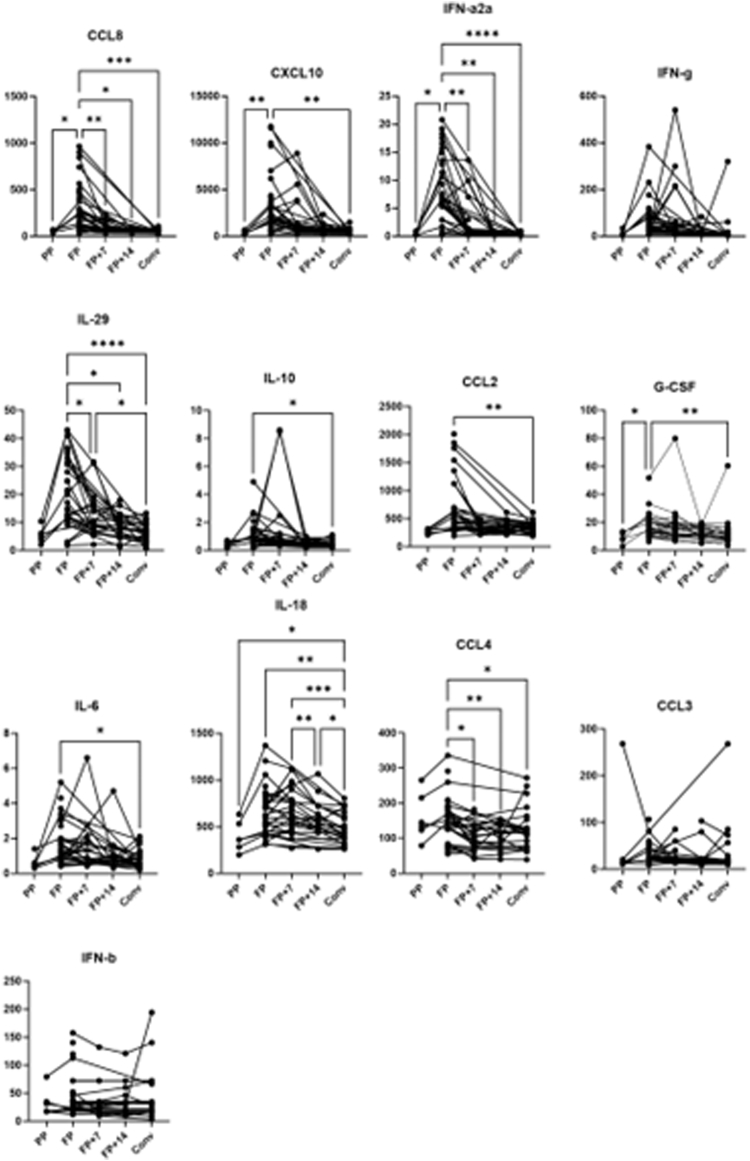
**Serum cytokine dynamics**. Change in serum cytokine concentration over time in PCR-positive contacts. Asterisks denote significant differences between time points according to Mixed effects analysis with Tukey’s multiple comparison test. ∗p < 0.05, ∗∗ p < 0.01, ∗∗∗p < 0.001, ∗∗∗∗p < 0.0001. **A.** Cytokines that were differentially expressed between PCR-positive and PCR-negative contacts. **B.** Cytokines that were not significantly differentially expressed between PCR-positive and PCR-negative contacts.

We next measured serum cytokine responses and compared associations with BMI and outcome. Paired serum cytokine, PCR and serology data was available from 69 INSTINCT COVID-19 contacts, 37 of whom became PCR-positive and 32 of whom remained PCR-negative and seronegative throughout the study (Figure S5). Paired NLF and serum cytokine data was available for 21 individuals. As in NLF, IFNα2, γ and λ, IL-6, CCL2 and 8, and CXCL10 were significantly upregulated at FP in the serum (Fig. 3A). IFNβ, IL27, CCL3, CCL4 were upregulated in the NLF but not the serum, whilst IL-10 and -18 and G-CSF were upregulated in the serum but not the NLF at FP. IFNγ and λ, CCL8, CXCL10 and IL-10 remained upregulated in PCR-positive contact serum at FP + 7 (Fig. 3A). The dynamics of serum cytokines were more consistent between individuals compared to NLF (Figure S6).

IFNα2 was the only serum cytokine to correlate significantly negatively with FP VL (Fig. 3B). Serum concentrations of IFNα2 and λ as well as CCL2 and 8, CXCL10, and G-CSF correlated positively with peak systemic symptom score. Serum IFNα2 and CCL2 also correlated positively with symptom burden score. Serum cytokine concentration was also positively associated with symptom outcomes at FP + 7; CCL8 concentration correlated significantly with symptom burden score (Fig. 3B). Whilst FP + 7 concentrations of 10 of 11 infection-associated cytokines in NLF correlated significantly positively with VL, in serum only G-CSF and IL-6 were significantly positively correlated with viral load (Fig. 3B).

**Figure S7:**
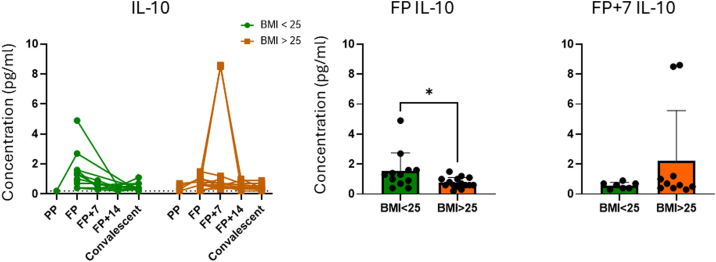
**Association between serum IL-10 dynamics and BMI group**. PCR-positive contacts were categorized into two BMI groups (BMI < 25 and BMI > 25). Serum IL-10 trajectories were plotted over time, with green lines representing contacts with BMI < 25 and orange lines representing those with BMI > 25. Differences in cytokine expression between BMI groups at FP and FP + 7 were assessed using the Mann-Whitney U test. ∗p < 0.05.

**Figure S8:**
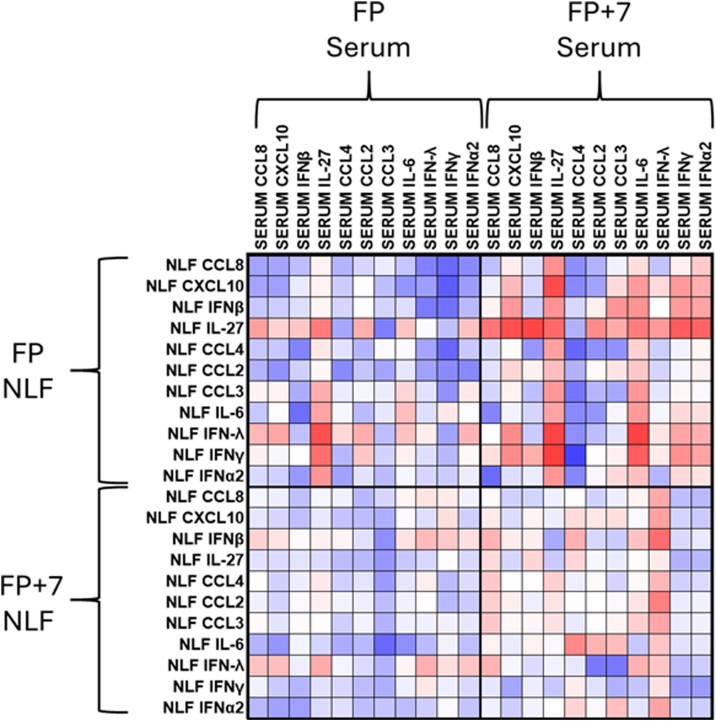
**Associations between serum and NLF cytokine concentrations**. Spearman’s correlation matrices of correlations between serum and NLF cytokine concentration at the FP and FP + 7 timepoints. Colours represent Spearman’s r values. Positive correlations are denoted by blue, negative correlations are denoted by red.

In contrast to observations in the airway mucosa, BMI did not correlate with serum cytokine responses except for IL-10 which correlated significantly negatively with BMI at FP (Fig. 3B). Unlike nasal cytokines, there were no significant differences in serum concentrations of CCL8, CXCL10, IFNβ, and IFNγ between BMI groups at FP or at FP + 7 (Fig. 3C). In concordance with the correlative association between BMI and serum IL-10, we observed significantly higher serum IL-10 concentrations in those with BMI < 25 at FP but not FP + 7 (Figure S7). Serum and NLF cytokine concentrations were not closely correlated, suggestive of compartmentalisation of immune responses (Figure S8).

### Nasal IgA titre is associated with nasal cytokine concentration and correlates with lower viral load

IgA is the most abundant antibody isotype at mucosal surfaces.^16^ SARS-CoV-2-specific IgA can limit viral replication, but it is still unclear how the speed and magnitude of *de novo* URT IgA induction relate to clinical and virological outcomes during a new SARS-CoV-2 infection.^17^, ^18^, ^19^ MSD V-PLEX assays were used to quantify nasal IgA specific to SARS-CoV-2 proteins (SARS-CoV-2 Nucleocapsid, SARS-CoV-2 whole S, SARS-CoV-2 S1 NTD, SARS-CoV-2 S1 RBD) as well as Spike proteins from common cold human coronavirus (HCoV) (HCoV-229E S, HCoV-NL63 S, HCoV-OC43 S, HCoV1-HKU1 S). Paired nasal IgA, PCR and serology data was available from 62 INSTINCT COVID-19 contacts, 42 of whom became PCR-positive and 20 of whom remained PCR and seronegative throughout the study. Of the 42 PCR-positive contacts, 32 had paired serum and NLF cytokine measurements. Of the 20 PCR-negative contacts, 12 had paired serum and NLF measurements (Figure S5).

**Fig. 4:**
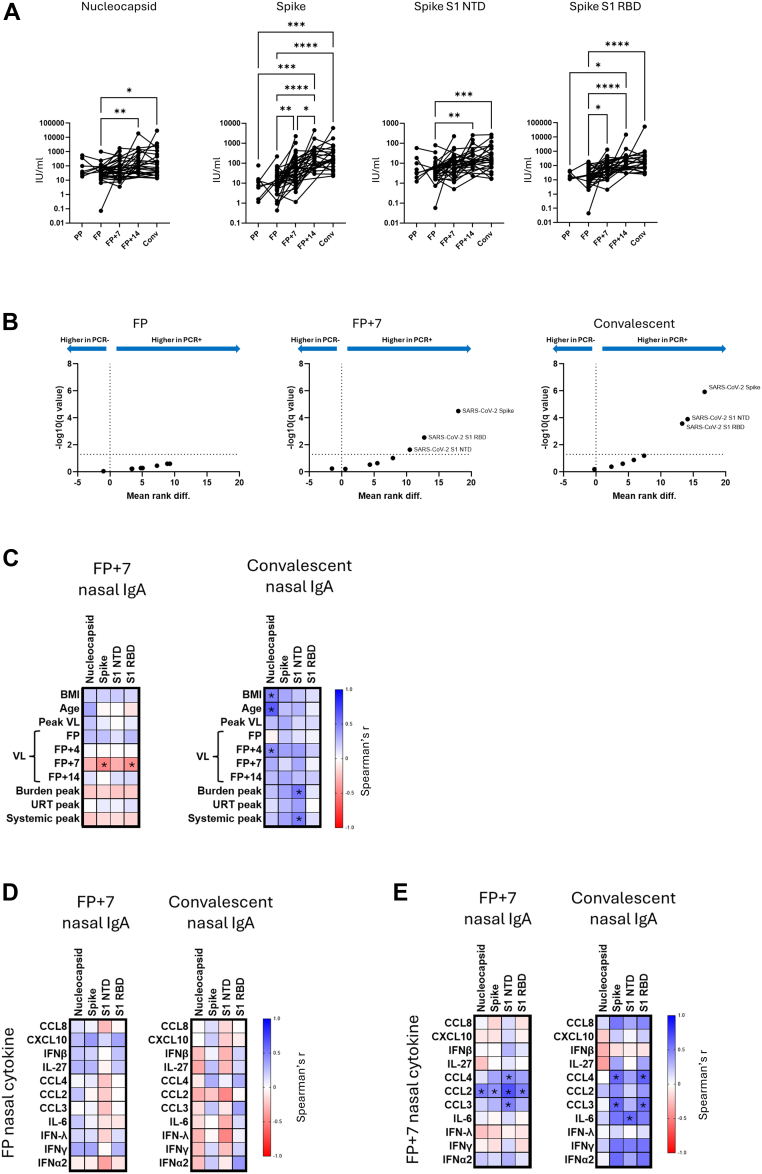
Infection-induced nasal IgA confers protection and correlates with nasal cytokine production. **A.** Change in nasal IgA titre over time in PCR-positive contacts. Concentrations (International Units (IU)/ml) of IgA specific to the indicated SARS-CoV-2 antigens are displayed. Asterisks denote significant differences between time points according to Kruskal–Wallis test with Dunn's multiple comparisons test. ∗adjusted p < 0.05, ∗∗adjusted p < 0.01, ∗∗∗adjusted p < 0.001, ∗∗∗∗adjusted p < 0.0001. **B.** Volcano plots depicting mean ranked difference outputs of multiple Mann Whitney U tests with two-stage step-up Benjamini, Krieger and Yekutieli FDR cut-off of 5% comparing concentrations of 4 SARS-CoV-2 antigens and 4 common cold human coronavirus S antigens in nasal lining fluid between PCR-positive (n = 42) and PCR-negative (n = 20) groups at the indicated time points. Analytes that were significantly different between groups (q < 0.05) are annotated. **C.** Spearman's correlation matrices of correlations between nasal concentrations of infection-associated IgA (defined in Fig. 4B) and the indicated demographic factors and outcome measures in PCR-positive contacts at the indicated time points. Colours represent Spearman's r values. Positive correlations are denoted by blue, negative correlations are denoted by red. Correlations with unadjusted p < 0.05 are indicated with an asterisk. Sample availability at each timepoint is displayed in Figure S5. **D.** Spearman's correlation matrices of correlations between FP infection-associated nasal cytokine concentration in PCR-positive contacts nasal concentrations of infection-associated IgA at FP + 7 (n = 21) and convalescence (n = 15) (Figure S5 displays overlap in participants with available nasal cytokine and nasal IgA data). Colours represent Spearman's r values. Positive correlations are denoted by blue, negative correlations are denoted by red. Correlations with unadjusted p < 0.05 are indicated with an asterisk. **E.** Spearman's correlation matrices of correlations between FP + 7 infection-associated nasal cytokine concentration in PCR-positive contacts nasal concentrations of infection-associated IgA at FP + 7 (n = 22) and convalescence (n = 13). Colours represent Spearman's r values. Positive correlations are denoted by blue, negative correlations are denoted by red. Correlations with unadjusted p < 0.05 are indicated with an asterisk.

Significant increases in all SARS-CoV-2-specific nasal IgA titres were observed between FP and convalescence in infected contacts. Spike and S1 RBD-specific IgA concentrations appeared to increase most rapidly, with significant increases in concentration evident between FP and FP + 7 (Fig. 4A). Consistent with these findings, we observed no significant differences in titres of any measured IgA between PCR-positive and PCR-negative contacts at FP, whereas at FP + 7 and at convalescence PCR-positive contacts had significantly higher titres of IgA recognising SARS-CoV-2 whole Spike, RBD and NTD. No significant differences in HCoV-specific IgA titres between PCR-positive and PCR-negative contacts were observed (Fig. 4B).

N-specific IgA titre correlated positively with both BMI and age at convalescence, but no other significant correlations between IgA concentration and demographic variables were observed (Fig. 4C). At FP + 7, S- and S1 RBD-specific IgA titres correlated negatively with viral load. Convalescent IgA titres specific to S1 NTD associated with higher prior viral load and symptom burden (Fig. 4C). FP nasal cytokine concentrations did not predict later SAR-CoV-2-specific IgA induction; no significant correlations were observed between FP nasal cytokine concentration and IgA concentrations at either FP + 7 or convalescence (Fig. 4D). However, FP + 7 concentrations of CCL2, CCL3 and CCL4 correlated significantly positively with titres of nasal anti-SARS-CoV-2 IgA (to spike, NTD and RBD) at the same time point. FP + 7 concentrations of CCL3 and 4 also correlated positively with S and S1 RBD-specific nasal IgA at convalescence (Fig. 4E).

## Discussion

Our longitudinal, prospective household contact study design enabled recruitment of close contacts of COVID-19 cases during ongoing exposure. We were able to collect URT samples from infected contacts very early post-infection, facilitating analysis of early mucosal and systemic immunological responses that associate with clinical and virological outcomes of infection. The immunologically naïve status of our cohort was essential for resolving early innate immune responses without the confounding effect of pre-existing adaptive immunity, which varies widely between individuals and has the potential to obscure protective innate immune signal. Given that the vast majority of the human population is now SARS-CoV-2 antigen experienced, studies of this kind are no longer feasible, underscoring the value of the observations reported here in understanding the role of local innate immunity in the context of a newly emerging pandemic respiratory virus.

We found that levels of nasal cytokines rapidly increased after infection, as recently observed in controlled human challenge studies.^8^ Concentrations of these nasal cytokines weakly but negatively correlated with subsequent viral load, suggesting a protective effect. This relationship was not observed one week post-infection when cytokine concentration positively correlated with concomitant viral load, consistent with higher viral loads driving more cytokine production, as previously reported.^20^ We also observed a negative correlation between early nasal cytokine expression and BMI which was not conserved at later timepoints and was not observed in serum cytokines. Together, these findings are consistent with elevated BMI specifically blunting or delaying early airway mucosal immune responses to SARS-CoV-2 infection.

Given our finding that elevated BMI is associated with protracted viral shedding and higher symptom scores, it is plausible that a BMI-associated delay in nasal cytokine production contributes to impaired early control of upper respiratory tract viral replication, resulting in more prolonged infection and increased disease burden. Although this interpretation is consistent with our observations, the observational nature of our study precludes direct mechanistic testing. Accordingly, the mechanistic basis for the delayed nasal mucosal, but not systemic, cytokine responses observed in individuals with elevated BMI remains to be determined.

One potential explanation is that increased adiposity leads to chronic low-grade inflammation at mucosal surfaces, which may alter epithelial barrier function and local innate immune signalling. This hypothesis is supported by experimental evidence showing that obese mice exhibit delayed type I interferon production in the respiratory tract following influenza virus challenge, and that primary human airway epithelial cells derived from obese individuals mount a delayed pro-inflammatory cytokine response following *in vitro* influenza virus stimulation.^21^^,^^22^ In addition, influenza virus challenge studies in obese individuals have demonstrated impaired respiratory mucosal innate immune cell interferon responses, which were linked to pre-existing metabolic alterations, including increased respiratory tract leptin levels.^23^ Evidence that elevated leptin disrupts innate immune signalling is provided by data from mice treated with exogenous leptin prior to influenza infection. Leptin treatment of these mice is sufficient to induce suppressor of cytokine signalling (SOCS) protein expression, negative regulators of JAK–STAT signalling, causing impairment of early interferon responses.^23^ Together, these findings suggest that chronic inflammation and metabolic differences associated with elevated BMI may selectively impair the induction of type I and III interferons and downstream cytokine production at the respiratory mucosa.

Our observation that elevated BMI associates with higher symptom scores aligns with the considerable body of evidence identifying high BMI as an independent risk factor for severe COVID-19 outcomes. Meta-analyses have shown that obesity roughly doubles the odds of hospitalisation, ICU admission and death.^2^^,^^24^ Findings from our cohort show that strong associations between BMI and COVID-19 outcome are present in clinically mild ambulatory COVID-19 cases and that even modest increases in BMI predispose to greater symptom burden in this population. The fact that the observed associations between BMI and both early innate immune responses and clinical and virological outcomes of SARS-CoV-2 infection pertain even at modestly elevated levels of BMI has important public health implications at the population level given that approximately 40% of the global population is overweight.^25^^,^^26^ Moreover, the higher viral loads and slower viral clearance observed in individuals with elevated BMI have been shown to associate with higher infectiousness and transmission risk.^27^

In addition to BMI affecting clinical outcome in infected individuals, elevated BMI has also been linked with a higher risk of SARS-CoV-2 infection. However, to date this association has been based on large epidemiological studies that do not measure exposure and may therefore be confounded by reduced testing in those with lower BMI because they are more often asymptomatic. In contrast, our cohort comprised infected and uninfected contacts with similar levels of documented SARS-CoV-2 exposure. We found that BMI was significantly higher in PCR-positive compared to PCR-negative contacts, supporting elevated BMI increasing the risk of infection with SARS-CoV-2 following exposure. However, given the small magnitude of the difference between BMI groups in our analysis and the substantial overlap of their confidence intervals, this finding should be interpreted with appropriate caution.

The mechanism by which elevated BMI affects susceptibility to infection is unclear, though it is possible that it relates to our observation that elevated BMI is associated with delayed initiation of local mucosal innate immune responses. Rapid innate immune gene upregulation has previously been observed in a subset of household contacts of COVID-19 cases that resist infection, and similar genes are rapidly and transiently significantly upregulated in experimentally SARS-CoV-2 challenged volunteers that resist infection but not in those who become infected. These studies support the concept that rapid innate immune activation can limit development of sustained replicative infection and hence BMI-associated retardation of these early immune responses might well contribute to elevated infection risk.^11^

Despite elevated BMI associating with delayed nasal cytokine responses, we did not observe associations between high BMI and delayed or lower-magnitude early nasal antibody induction. Conflicting evidence including both negative and positive associations between BMI and humoural responses to SARS-CoV-2 has been reported in the literature.^28^, ^29^, ^30^, ^31^ Delayed early local immune responses, as observed in individuals with higher BMI in this study, could lead to higher viral and antigen loads which in turn drive stronger adaptive responses. This would be consistent with our observation of a positive correlation between BMI and convalescent nasal IgA titre and other observations of higher-magnitude antibody responses in those with higher BMI.^30^

Around 1-week post-infection, the earliest time point at which SARS-CoV-2-specific mucosal IgA was significantly elevated, we observed a negative correlation between IgA levels and viral load suggesting that a stronger IgA response within the first week of infection may contribute to superior control of viral replication. Additionally, nasal chemokine concentration 1-week post-infection correlated with both concomitant and convalescent nasal IgA titre. This suggests a coordinated innate and adaptive local immune response to SARS-CoV-2 infection in which nasal chemokines have a role in promoting stronger nasal antibody production.

Our relatively small sample size reflects the practical challenges associated with recruiting very recent COVID-19 contacts with well-characterised SARS-CoV-2 exposure and close longitudinal follow up. Additionally, whilst helpful for generating clinically relevant insights utilising a thresholding approach to analysis based on a pre-specified BMI value of 25 causes omission of information by essentially converting continuous variables to categorical data. As such our findings should be validated in larger cohorts and interpreted alongside our correlative analyses of continuous data. Another limitation is that viral load was quantified by E gene PCR which quantifies viral RNA as opposed to replication-competent virus. Assays targeting subgenomic RNA (sgRNA) have been proposed as markers of active replication, their specificity is limited by reports of sgRNA persistence after culturable virus is no longer detectable and indistinguishable decline rates compared to genomic RNA within individuals over time,^32^ reducing their interpretability in clinical samples. Definitive assessment of infectious virus by plaque assay was not feasible here because many samples have subsequently been used for additional assays or undergone freeze–thaw cycles that compromise culturability. Notwithstanding these limitations, prior work in the INSTINCT cohort demonstrates a reasonable correlation between qRT-PCR E gene-based viral load measurement and infectious virus, supporting the use of our approach to measure viral load in this community setting.^33^

Future studies could also aim to characterise metabolic features such as leptin levels that may contribute to explaining the observed association between high BMI and impaired local immune responses.^6^^,^^34^ Furthermore, it would be interesting to determine whether the observed association between BMI and early kinetics of nasal cytokine response are specific to COVID-19 or also pertain to other respiratory infections.

Our findings provide evidence for a role of URT mucosal immune responses, particularly nasal chemokine and SARS-CoV-2-specific IgA induction, in limiting viral load. We observed that higher BMI is associated with significantly delayed induction of mucosal cytokine responses which may contribute to suboptimal viral control and unfavourable clinical outcomes, even in mild, ambulatory illness. These results reinforce the importance of population-wide public health messaging to avoid even modest increases in BMI above normal levels. More broadly, our findings underscore the need for tailored interventions that enhance early URT innate immune responses, particularly in individuals at higher risk due to elevated BMI.

## Contributors

INSTINCT study group investigators contributed to participant recruitment and enrolment, collection and biobanking of biological samples, and management, administrative and logistical aspects of the INSTINCT study.

Conceptualisation: JF, RK, AL.

Software: JF, RK, JSN, EC, AK.

Validation: JF, EC, AK.

Formal analysis: JF, JSN, RK, EC, AK.

Investigation: JF, RK, JSN, EC, AK, SH, ND, SN, MTW, JJ, KM, SB, TDP, RV, CL, GT, JD, JT, AL.

Data curation: JF, RK, JSN, EC, AK.

Writing—original draft: JF, RK, JT, AL.

Writing—review & editing: JF, RK, JSN, EC, AK, SH, ND, SN, MTW, JJ, KM, SB, TDP, RV, CL, GT, JD, JT, AL.

Visualisation: JF, EC, AK.

Supervision: JF, RK, GT, JD, JT, AL.

Project administration: AL.

Funding acquisition: RK, JD, AL.

The following authors accessed and verified that data underlying data presented in this manuscript: JF, EC and AK.

All authors have read and approved the final version of this manuscript.

## Data sharing statement

Anonymised, de-identified versions of datasets used to produce graphs and tables presented in this manuscript are available with investigator support upon reasonable request to the corresponding author (jfenn@ic.ac.uk) following publication. INSTINCT study documents including study protocol, template participant case record form (CRF), informed consent form and participant information sheet (PIS) are also available upon request.

## Declaration of interests

The authors declare no competing interests.
