# Supplementary material for: Elevated body mass index is associated with delayed protective airway mucosal immune responses in mild SARS-CoV-2 infection
Source: eBioMedicine. 2026 Mar 27;126:106215. doi: 10.1016/j.ebiom.2026.106215 (PMC13058998; doi:10.1016/j.ebiom.2026.106215)
Supplement: Supplemental Tables S1 and S2 [file mmc1.pdf]

Type	Symptom diary question	Answers (points)
	In the last 24 hours, have you…	
Canonical definition	felt feverish?	Yes (1.5) / No (0)
	had a persistent cough lasting for more than 4 hours?	Mild (1) / Moderate (2) / Severe (3) / No (0)
	coughed up phlegm or mucous?	Yes (1.5) / No (0)
	had loss or change of smell or taste?	Yes (1.5) / No (0)
Lower Respiratory Tract	felt unusually breathless?	Mild (1) / Moderate (2) / Severe (3) / No (0)
	had a wheeze?	Yes (1.5) / No (0)
	coughed blood?	Yes (1.5) / No (0)
	had chest pain?	Mild (1) / Moderate (2) / Severe (3) / No (0)
Upper Respiratory Tract	had a runny or blocked nose?	Yes (1.5) / No (0)
	had a sore throat?	Mild (1) / Moderate (2) / Severe (3) / No (0)
	had red or irritated eyes?	Mild (1) / Moderate (2) / Severe (3) / No (0)
	had a hoarse voice?	Yes (1.5) / No (0)
Gastrointestinal	had unusual tummy pains?	Yes (1.5) / No (0)
	had loss of appetite?	Mild (1) / Moderate (2) / Severe (3) / No (0)
	felt sick or vomited?	Mild (1) / Moderate (2) / Severe (3) / No (0)
	had diarrhoea?	Mild (1) / Moderate (2) / Severe (3) / No (0)
Systemic	had a headache?	Mild (1) / Moderate (2) / Severe (3) / No (0)
	had unusual muscle aches?	Mild (1) / Moderate (2) / Severe (3) / No (0)
	felt tired or generally unwell?	Mild (1) / Moderate (2) / Severe (3) / No (0)
	been confused?	Yes (1.5) / No (0)

Supplemental Table 1 - Daily symptom diary questionnaire. Contribution of each symptom to the symptom burden score is detailed in the third column. The total burden score is calculated as the summed scores of all present symptoms, divided by the maximum possible score (46.5). Body domain scores, e.g. systemic burden score, are calculated using scores only from the relevant section divided by the maximum possible score (in the case of systemic symptom burden 10.5).

	Total	PCR+<br>(Exposed infected)	PCR-<br>(Exposed uninfected)
**Contacts**	54	39	15
**Female (%)**	28 (51.9%)	17 (43.6%)	11 (73.3%)
**Median Age (IQR)**	31 (24.75)	34 (19.5)	20 (29.5)
**Median BMI (IQR)**	24.2 (5.7)	25.8 (4.3)	22.5 (4.4)
**Median HH size (IQR)**	3 (2)	3 (2)	4 (2.5)

Supplemental Table 2 - Baseline demographic and clinical characteristics of household contacts exposed to PCR-confirmed SARS-CoV-2 primary cases. Number of female contacts and associated percentages as well as medians and interquartile ranges (IQR) of age, BMI and household (HH) size are displayed for PCR-positive and PCR-negative contacts used in the analyses displayed in figures 1 and 2.
